# “Gloppiness” Phenomena and a Computer Vision Method to Quantify It

**DOI:** 10.3390/gels9070532

**Published:** 2023-06-30

**Authors:** Shijian Wu, Mark Mintel, Baran Teoman, Stephanie Jensen, Andrei Potanin

**Affiliations:** 1 Colgate-Palmolive Company, Piscataway, NJ 08854, USA; 2 Department of Chemical and Biochemical Engineering, Rutgers University, Piscataway, NJ 08854, USA

**Keywords:** gloppiness, rheology, computer vision, rupture time, python

## Abstract

In this study, we present a rapid, cost-effective Python-driven computer vision approach to quantify the prevalent “gloppiness” phenomenon observed in complex fluids and gels. We discovered that rheology measurements obtained from commercial shear rheometers do show some hints, but do not exhibit a strong correlation with the extent of “gloppiness”. To measure the “gloppiness” level of laboratory-produced shower gel samples, we employed the rupture time of jetting flow and found a significant correlation with data gathered from the technical insight panelist team. While fully comprehending the “gloppiness” phenomenon remains a complex challenge, the Python-based computer vision technique utilizing jetting flow offers a promising, efficient, and affordable solution for assessing the degree of “gloppiness” for commercial liquid and gel products in the industry.

## 1. Introduction

Rheology modifiers are essential additives in the manufacture of personal care, oral care, and cosmetic products. They play a crucial role in determining the rheological properties of a product, such as viscosity, shelf stability, ease of application, and other key indicators [1,2]. Versathix (CRODA company) is a commercial product typically used in cosmetic formulations as a thickening agent. This product is a mixture of PEG-150 pentaerythrityl tetrastearate, PPG-2 hydroxyethyl cocamide, and Aqua. Xanthan gum (XG) (CPKelco Company) is a complex polysaccharide used as a food additive and a viscosity modifier in many industries. Its molecular weight is usually very high, in the millions of Daltons(or g/mol). However, the exact molecular weight can vary widely depending on the specifics of its production. Both of them are commonly used rheology modifiers in the personal care industry. Versathix is a mixture of associative polymers that contain sticky groups that form a gel network through weak chemical or physical interactions with surfactants [3,4]. On the other hand, xanthan gum is capable of forming highly viscous solutions or gels even at low concentrations [5].

A thorough understanding of the dynamics of complex fluids and gels, such as liquid hand soap and shower gels, is crucial for ensuring the quality, safety, and overall performance of these products. One particular aspect of their behaviors, known as “gloppiness”, describes their tendency to become distorted, discontinuous, swollen, ruptured, or broken into pieces when subjected to stress, such as when they are pulled or squeezed out of containers. This phenomenon of gloppiness is particularly prevalent in surfactant-based micellar fluid and gel systems. Surfactants are compounds that lower the surface tension between two substances, such as oil and water, and are commonly used in soaps, detergents, and personal care products. The micellar structures that form within these systems can exhibit complex behavior, and their response to stress can lead to challenges for both manufacturers and consumers.

Due to these challenges, it is crucial to gain a more in-depth understanding of the underlying causes and mechanisms of the “gloppiness” phenomena. This knowledge will help develop improved manufacturing processes and product formulations, ensuring that the end products maintain their intended quality and effectiveness, while also minimizing waste and enhancing overall customer satisfaction.

In order to address these challenges, we introduce a jet flow computer vision technique to investigate the dynamics of complex fluids and gels. A jet refers to the flow of fluid that exits a nozzle into an ambient fluid with differing velocities. The nozzle helps regulate pressure, which subsequently controls the jet’s velocity. The jet flow method simulates the squeezing process by employing a syringe pump to manage the flow rate of the sample passing through the nozzle, while a high-speed camera captures the jet flow evaluation process for various samples at an appropriate frame rate. Subsequently, a Python-based computer vision algorithm analyzes the recorded flow video.

## 2. Materials and Methods

### 2.1. Materials

In this study, six laboratory-made shower gel samples were evaluated. These samples were divided into two groups, with three samples in each group. One group was formulated using versathix as the thickener, while the other group was formulated using xanthan gum. The reason for selecting these two thickening agents is that they are commonly used in various personal care, home care, and oral care products as rheology modifying agents in the daily chemical industry. They also represent two main types of rheological behavior that are typical for personal care products: viscoelastic fluids and gel-like products with yield stress. These model samples serve as ideal starting points for investigating “gloppiness” issues.

Table 1 displays the composition of the three shower gel samples that utilize Versathix as the thickener. The degree of gloppiness in these samples is categorized as low, medium, and high, depending on the concentration of Versathix, which ranges from 0.2%, 0.4%, and 0.6%, respectively. Table 2 shows the chemical composition of the three shower gel samples that use xanthan gum as the thickener. The degree of gloppiness in these samples is also categorized as low, medium, and high, depending on the concentration of xanthan gum, which varies from 0.9%, 1.3%, and 1.8%.

### 2.2. Shear Rheology Tests

#### 2.2.1. Flow Curve

The flow curve of the shower gel samples was determined using a TA ARES 2 rheometer. The shear viscosity (η) of the samples was analyzed as a function of shear rate ($\dot{\gamma }$) in the range of $\dot{\gamma }$ = 0.01 (s${}^{-1}$) to $\dot{\gamma }=1000$.

#### 2.2.2. Small Amplitude Oscillatory Shear (SAOS)

The small amplitude oscillatory shear (SAOS) test was carried out using the TA ARES 2 rheometer. The test measured the elastic modulus ($G^{\prime }$ [Pa]) and the loss modulus ($G^{\prime \prime }$ [Pa]) as a function of angular frequency (ω [rad/s]) over a range of ω values from 0.01 [rad/s] to 1000 [rad/s]. The results were then fitted to the Maxwell viscoelastic model to determine the number of modes (m) and the Maxwell shear relaxation time ($\tau_{Ri}$).

### 2.3. “Gloppiness” Degrees Evaluations by Trained Panelists

The degree of “gloppiness” of the laboratory-made shower gel was evaluated by a team of well-trained technical insight panelists. Each sample was evaluated by 5 different panelists at home at room temperature. The evaluation process followed a documented standard operating procedure, which is specific to the assessment of shower gel and personal care products. The “gloppiness” degree ($G_{0}$) was translated into standardized sensory attributes for both visual and tactile properties.

The visual properties also included appearance, flow speed, flow evenness, dispensing gloppiness, consistency, and ability to hold shape. The tactile properties included the characteristics of breaking apart and springing back and sliminess. The panelists rated each attribute on a scale of 0 to 10, with the final score for each attribute being the average of all panelists. To eliminate bias, all samples were given three-digit codes during the panel tests. A higher score for an attribute indicated a stronger presence of that attribute, and vice versa.

**Figure 1:**
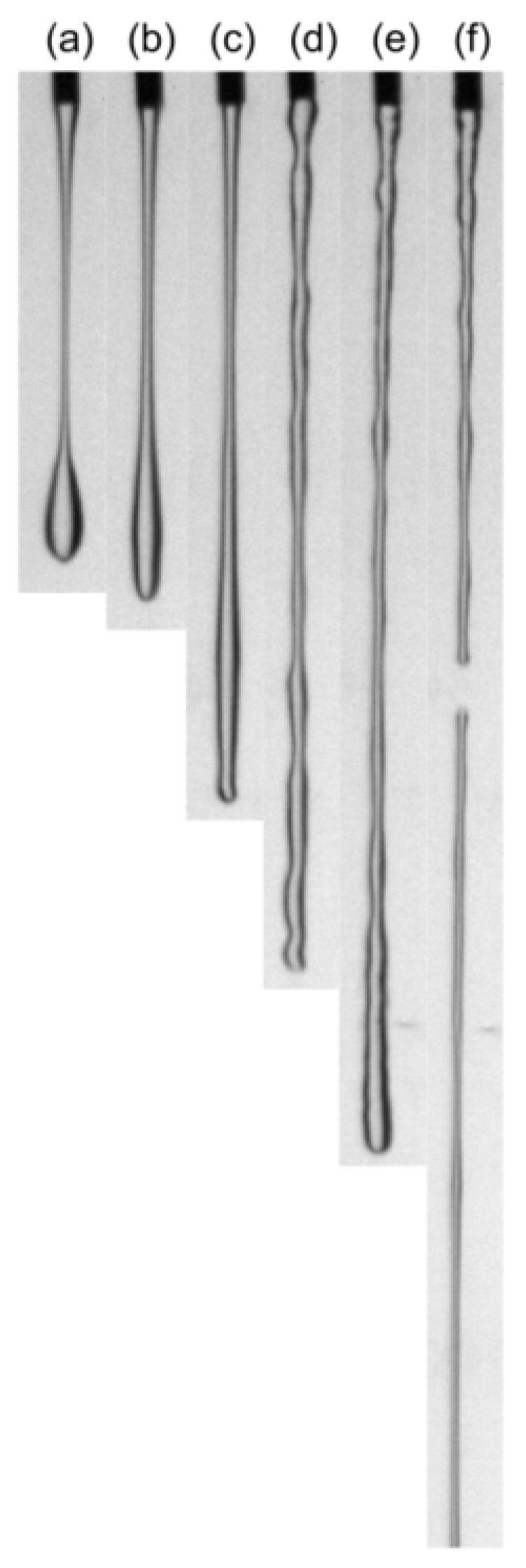
Jetting flow filament of the six shower gel samples (**a**–**f**) are XG-LG, XG-MG, XG-HG, Versathix-LG, Versathix-MG, and Versathix-HG.

The key sensory attributes are listed in Table 3. The results showed no significant correlation between the “gloppiness” degree of a sample and other sensory attributes, such as consistency and ability to hold shape. This was further demonstrated in the plot shown in Figure 1.

### 2.4. Jetting Flow Test

**Figure 2:**
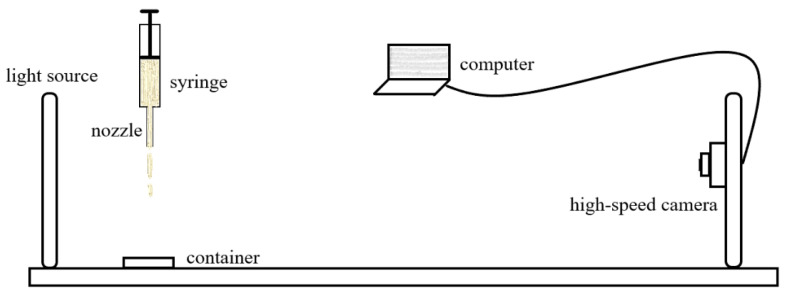
Schemetic of Jetting flow setup.

Figure 2 showed the schematic of the house-built jet flow setup that was used for the “gloppiness” degree test of the shower gel samples. In a typical experiment, the sample was pushed out from the nozzle with a 1.70 mm inner diameter and a length of 1 inch by a syringe pump with a flow rate of 200.00 mL/h. A container was put under the syringe nozzle to hold the jetted shower gel samples from the nozzle.

The setup was selected so as to mimic the actual consumer operation. With the flowrate of 200 mL/h, and a needle with 1.7 mm inner diameter, the pressure drop (ΔP) from one end of the pipe to the other is given by the Poiseuille law as follows:

(1) $$\Delta P=8\eta LQ/(\pi r^{4})$$

where:

ΔP is the pressure difference,
η is the dynamic viscosity of the fluid,
*L* is the length of the pipe,
*Q* is the volumetric flow rate, and
*r* is the radius of the pipe.

Assuming viscosity being close to glycerine at room temperature, 1.5 Pa.s, for the conditions of our tests this gives ΔP = 3 MPa. For the flow in typical real-user conditions: the flow rate is around 5 mm/s, the diameter of the bottle hole is 8 mm, the thickness of the hole is around 2 mm, and the viscosity of the shower gel is similar to glycerin as 1.5 Pa.s. This gives about the same pressure drop.

A high-speed camera (FASTEC IL5) was used to record the jetting process of the shower gels. The recorded video was then processed by using a computer vision Python algorithm to extract useful information such as the drop size, jetting length, breakage/rupture time, and thinning dynamic of the jetting filament. It should be noted that one can adjust the field of view (FoV) by zooming in or zooming out the high-speed camera to focus on the location of interest. The processes of extracting information is as follows:

- Record the videos of the jet experiment with a high-speed camera. One needs to carefully adjust the frame rate (FPS) and the field of view (FoV) to get a suitable video for future data analysis.
- Inputs the video to a Python-based algorithm. The packages used here are OpenCV, Numpy, and Pandas.
- Applied OpenCV canny function to detect the edge of the jet in the video. The details of the canny function can be found in this link: https://docs.opencv.org/3.4/da/d22/tutorial_py_canny.html (accessed on 1 March 2023)
- Save the edge position for the whole video as a panda DataFrame. The details about the panda DataFrame can be found in this link: https://www.geeksforgeeks.org/pandas-tutorial/ (accessed on 1 March 2023)
- Find the local and global minimum value for each frame. The reason that we also want to find the local minimum is that the global minimum is not always the breakage(rupture) location.
- Select the right local and global minimum value for each frame with the Algorithm. Normalize it and plot it as shown in Section 3.2.

## 3. Results

### 3.1. Shear Rheology

#### 3.1.1. Flow Curve

**Figure 3:**
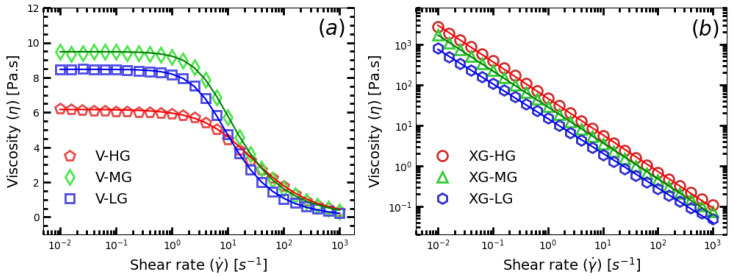
The shear viscosity ($\eta_{s}$) as a function of shear rate ($\dot{\gamma }$ ) for the (**a**) versathix based and (**b**) Xanthan Gum based shower gel samples.

The shear viscosity ($\eta_{s}$) as a function of shear rate ($\dot{\gamma }$) were measured for shower gel samples. The flow curve of the three versathix based samples are shown in Figure 3a. All three samples show a plate of viscosity when the shear rate ($\dot{\gamma }$) is relatively low ($\dot{\gamma }\leq 2\times 10^{-1}$ [s${}^{-1}$]), and a strong shear thinning behavior were found in these samples when the shear rate ($\dot{\gamma }$) is higher than a critical threshold ($\dot{\gamma }\geq 1\times 10^{0}$ [s${}^{-1}$]). The flow curve of xanthan based shower gel samples are shown in Figure 3b. These three samples show strong shear thinning behavior within the whole range of the tested shear rate ($10^{-2}\leq \dot{\gamma }\leq 2\times 10^{3}$ [s${}^{-1}$]). It is not difficult to find that the shear viscosity under the flow curve of the versathix based shower gel is quite different from the xanthan gum based shower gel samples. The reasons for that will be discussed in the Section 4.

#### 3.1.2. Small Amplitude Oscillatory Shear Test

Small amplitude oscillatory test is the most common method for measuring viscoelastic properties of soft materials by using a rotational rheometer. The linear viscoelastic rheology of micelles and polymers systems can be fitted to a multi-mode Maxwell model as in Equation (2) [6,7].

(2) $$G^{\prime }\left(\omega \right)=\sum_{i=1}^{n}\frac{G_{i}\omega^{2}\tau_{Ri}^{2}}{1+\omega^{2}\tau_{Ri}^{2}};\;G^{\prime \prime }\left(\omega \right)=\sum_{i=1}^{n}\frac{G_{i}\omega \tau_{Ri}}{1+\omega^{2}\tau_{Ri}^{2}},$$

**Figure 4:**
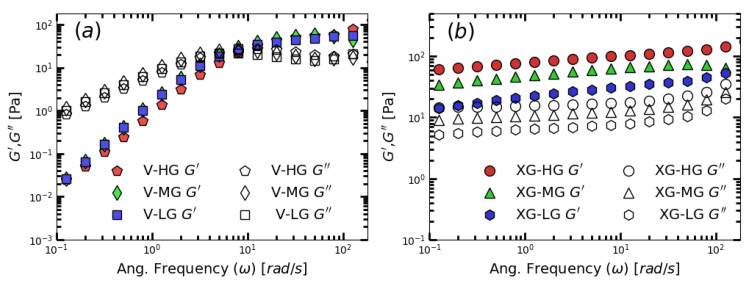
The elastic ($G^{\prime }$) and loss modulus($G^{\prime \prime }$) as a function of angular frequency (ω) for (**a**) Versathix- based and (**b**) xanthan gum based laboratory-made shower gel samples.

As shown in Figure 4a, The flow of Versathix based shower gel samples are dominated by viscosity at low angular frequency, while the elasticity becomes dominant as the angular frequency increases. These are typical SAOS tests results of micellar systems. By fitting the data to Maxwell model shown in Equation (2). We can calculate the maxium relaxation times ($\lambda_{s}$) for those samples as showed in Table 4. Figure 4b showed the SAOS tests for xanthan gum-based shower gel samples. The three samples showed that the elastic modulus ($G^{\prime }$) is always higher than the loss modulus ($G^{\prime \prime }$) within the range of the whole test angular frequency. These showed that the xanthan gum-based shower gel samples are soft gel [8]. The XG-based shower gel samples behaved close to a solid since the stress in the SAOS measurements was below the yield stress. It is not difficult to find that the effects of versathix and xanthan gum are quite different. Both of them can increase the viscosity of shower gel products, while versathix will enhance the viscoelastic behavior of the samples, and Xanthan gum would increase the yield stress of the samples.

### 3.2. Jet Flow of Complex Fluids

In the jetting flow experiment, a sample fluid was piped out from a needle with a diameter of 1.70 mm under gravity. The flow rate of the sample fluid was controlled by a syringe pump. During the experiment process, all of the six shower gel samples went through a filament thinning and finally breakage process when the flow rate is relatively small. As the flow rate increased, the xanthan gum-based sample shows a continuous flow, while however, the versathix-based samples became rough, distorted, and broken into pieces. These phenomena echo the previous statement that the versathix and xanthan gum-based samples are very different in the flow curve and SAOS section.

**Figure 5:**
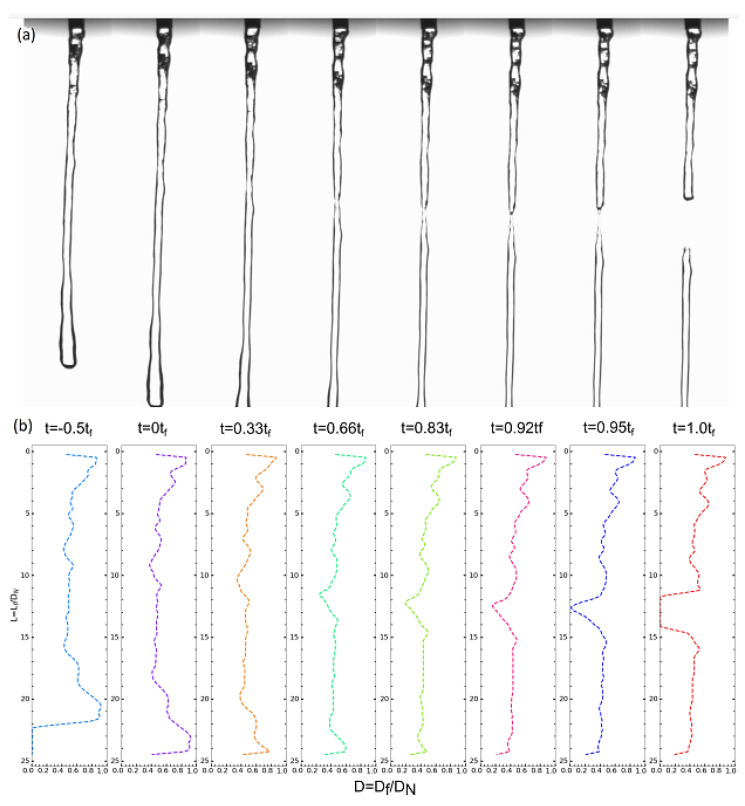
Jetting flow filament breakage process of MG-versathix samples. (**a**) the snapshots for the breaking process. (**b**) The diameter map of the filament at different time red points.

Figure 5 shows a demo of the filament breakage process of the versathix-based sample with a middle “gloppiness” degree. The diameter is not changed much from t=−0.5$t_{f}$ to t=0$t_{f}$, here, $t_{f}$ is the rupture time of the filament. Starting from t=0$t_{f}$, there is a local minimum diameter value formed quickly in the arrow-labeled location. During the rupture process, the versathix-based shower gel filament was not thinning in a smooth linear way, but the filament was ruptured in a way similar to solid rubber or other visco-elastic gel. Figure 5b is the 2D diameter map of detected by the python code in Figure 5a. $L_{f}$ is the filament length, $D_{f}$ is the filament diameter, $D_{N}$ is the diameter of the nozzle. It is easy to see that there is a minimal value starting around $t=0t_{f}$, and the minimal value decreases very fast as time goes on.

**Figure 6:**
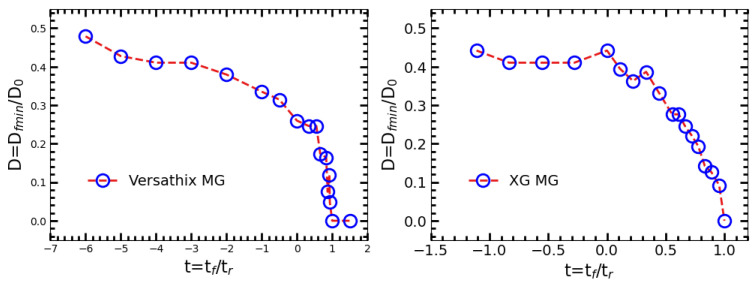
Normalized diameter ($D=D_{fmin}/D_{0}$) of demo sample as a function of normalized time $t=t_{f}/t_{r}$. Rupture point (t=0) was defined as the time point that the filament diameter start to accelerating decrease.

As shown in Figure 6, to better understand the filament rupture process, we plotted the normalized filament diameter ($D=D_{fmin/D_{0}}$) as a function of normalized time ($t=t_{f}/t_{r}$). Here $D_{fmin}$ is the minimum diameter of a filament at the respect time *t*. $D_{0}$ is the diameter of the nozzle used in the experiment. $t_{f}$ is the time point before or after the rupture time start, and $t_{r}$ is the rupture time of the sample. It can be found that the normalized minimum diameter *D* decrease slowly before the rupture point (t=0), while *D* decrease dramatically fast after the rupture point. The rupture time ($t_{r}$) is the time between the rupture point to the time the filament break (breakage point $t_{b}$).

**Figure 7:**
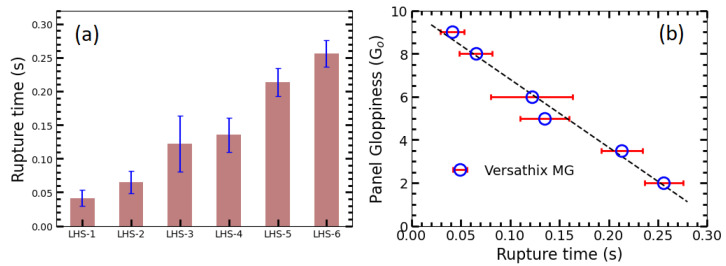
(**a**) Rupture time for the six shower gel samples. (**b**) The correlation of Panel Gloppiness degree with the Rupture time.

The same python-based visional analysis method was applied to study the filament behavior of the six shower gel samples. The rupture time $t_{r}$ of all those samples was plotted in Figure 7a. The experiments were repeated 6 times to get the statistic error bar. It can be found that the rupture time of the shower gel can be ranked as shown in Figure 7b, And there is a strong correlation ($R^{2}=0.98$) between the rupture time and the panel “gloppiness” ($G_{o}$).

## 4. Discussion

### 4.1. Shear Rheology

Shear rheology tests (Flow curve and SAOS) for the shower gel samples can show some hints of “gloppiness” of the product. For example, the flow curve in Figure 3 shows the shear viscosity (η) as a function of shear rate ($\dot{\gamma }$) of the samples. For versathix-based samples, the shear viscosity (η) is almost contant when shear rate ($\dot{\gamma }$) is relatively small ($\dot{\gamma }\leq 2\times 10^{-1}$ s${}^{-1}$), and it showed a strong shear-thinning behavior when the shear rate ($\dot{\gamma }$) was within the range 1 s${}^{-1}$$\leq \dot{\gamma }\leq$ 100 s${}^{-1}$. The Xanthan gum-based samples show very high shear viscosity at small shear rate ($\dot{\gamma }\leq 0.1$), but they show strong shear thinning behavior within the whole range of test with shear rate ($\dot{\gamma }$) within 0.1 s${}^{-1}$, $\dot{\gamma }\leq \leq$ $\;10^{2}$ s${}^{-1}$. The performance of real shower gel products can have similar viscosity profile as the versathix-based or the xanthan gum-based samples. However, it is difficult to directly use the flow curve methods to build a quantification model for “gloppiness”.

The small amplitude oscillatory sweep (SAOS) test shows the storage modulus ($G^{\prime }$) and the loss modulus ($G^{\prime \prime }$) as a function of angular frequency. As shown in Figure 4a, The flow of versathix-based shower gel samples is dominant by viscosity at low angular frequency, while the elasticity becomes dominant as the angular frequency increases. These are typical SAOS test results of micellar systems. By fitting the data to the Maxwell model shown in Equation (2). We can calculate the maxium relaxation times ($\lambda_{s}$) for those samples as showed in Table 1. Figure 4b showed the SAOS tests for xanthan gum based shower gel samples. The three samples showed that the elastic modulus ($G^{\prime }$) is always higher than the loss modulus ($G^{\prime \prime }$) within the range of the whole test angular frequency. These showed that the xanthan gum-based shower gel samples are soft gel [8]. The XG-based shower gel samples behaved close to a solid since the stress in the SAOS measurements was below the yield stress. It is not difficult to find that the effects of versathix and xanthan gum are quite different. Both of them can increase the viscosity of shower gel products, while versathix will enhance the viscoelastic behavior of the samples, and Xanthan gum would increase the yield stress of the samples.

### 4.2. The Effect of “Gloppiness”

For manufacturers, the gloppiness behavior of these fluids and gels can pose significant logistical challenges. As these products flow through pipes and transportation systems within manufacturing plants, their gloppiness can cause blockages or damage to equipment, leading to increased maintenance costs and potential disruptions to the production process. Furthermore, the quality control of products exhibiting gloppiness can be more difficult, as they may not display uniform properties throughout the entire batch.

From the consumer’s perspective, the clumpy structure of these gloppy fluids and gels can lead to waste and an overall less satisfactory experience. For example, when squeezing a gloppy shower gel out of a container, clumps may break off, making it harder to control the amount dispensed and leading to waste as the product does not spread evenly over the skin. Similarly, for shower gels, gloppiness may result in uneven application and an overall less satisfying user experience.

The dynamics of jet breakage are intricate and have garnered the attention of numerous research groups. Liquid jets, which are cylindrical-shaped liquid streams, break due to a variety of physical properties, including surface tension, density differences, gravitational interactions, viscosity, and non-Newtonian rheology [9]. Our jet flow breakage technique offers an affordable and efficient method for quantifying the ”gloppiness” degree of surfactant and associative polymer-based complex fluids.

By comparing the outcomes of our approach with those obtained from human evaluation panelists, our method has the potential to significantly reduce the cost and time needed to assess the “gloppiness” of shower gel products and other similar complex fluids.

In this study, we present a jetting flow technique as a potential rapid and cost-effective approach for measuring the degree of “gloppiness” in surfactant-based complex fluids and gels. Our findings indicate that the gloppiness degree of a product is inversely related to the rupture time ($t_{R}$) observed in a jetting or dripping flow at a flow rate of 200 mL/h and a nozzle inner diameter of 1.7 mm.

It is worthwhile to emphasize that the suggested jetting flow technique is understood here primarily as a simple way to emulate consumer perception of products to be used along with other emulative tests widely used in industry, such as shape retention [10]. It closely follows the actual human action thereby allowing to quantify perception as confirmed by comparison to panel ratings. The advantage of this technique is that it is applicable to products with different structure and different mechanical properties as will be confirmed by rheological measurements. However, establishing quantitative relation between rheological parameters of these fluids or their microstructure and their perceived “gloppiness” is beyond the scope of this work.

Traditionally, industrial companies have relied on trained panelists to assess various “sensorial attibutes” of consumer products such as shape retnetion, firmness etc. “Gloppiness” can be thought of as one of such attributes. However, these panel evaluations are both costly and time-consuming. While rheological measurements under shear, such as viscosity (η), elastic and loss modulus (G’, $G^{\prime \prime }$), and yield stress ($\sigma_{0}$) can provide some insight into the behavior of the product (see, e.g., [10]), it is difficult to quantify this phenomenon solely based on these measurements. There is a need for a more efficient and effective method to quantify the “gloppiness” of complex fluids and gels in the manufacturing and consumption of detergent and cosmetic products. Extensional rheology also provides important information, particularly relevant to the gloppiness attribute. In polymer physics, polymer liquids fracture like solids is widely discussed [11,12], and a somewhat similar problem of polymer rupture is often described in terms of the “time-to-break” of a melt [13,14], which may be used to discriminate between fluid and rubbery behavior. In the consumer products industry there is a need for a similar simple efficient tool to quantify “gloppiness” of complex fluids, such as shower gels.

### 4.3. Jetting Flow under Gravity

Jetting flow from a nozzle under gravity can mimic the squeezing process of shower gel products. The behavior of the jetting flow can be observed by combining the advantage of a high-speed camera and the python-based computer vision algorithm. It is hard to cover and explain all the behaviors of the jetting flows, but in our work, the rupture time can somewhat show the degree of “gloppiness” (Go) of a sample for the necessary of industrial applications. Rupture includes different kinds of breakage mechanisms such as necking, fracture, and breakup driven by surface tension.

Necking is a form of tensile deformation wherein a major part of the strain disproportionately localizes in a tiny region of the material. Due to plastic flow, the cross-sectional area of this region reduces, giving the “neck” its name. This process precedes ductile fracture in ductile materials, making it a more gradual and less abrupt process compared to fracture.

The fracture of materials is a sudden process that typically occurs in either a brittle or ductile way. Brittle fracture is sudden and dramatic, occurring without any prior plastic deformation and predominantly because of the rapid progression of cracks. Ductile fracture, in contrast, involves considerable plastic deformation and necking before the actual break, yet the event of fracture is still quick as it’s propelled by the swift release of accumulated elastic energy.

Breakup driven by the surface tension process is predominantly seen in fluids, where the liquid’s intermolecular forces create behavior akin to an elastic sheet. The liquid strives to reduce its surface area because of surface tension, leading to the formation and disintegration of droplets or jets. The breakup rate here usually relies on the fluid’s viscosity and inertia, making it a more predictable and smoother process compared to the sudden nature of fractures in solid materials.

The effect of surface tension was also considered in this work. In Newtonian fluids, which maintain constant viscosity regardless of the applied stress, filament breakup is chiefly regulated by competition between surface tension. However, the scenario with viscoelastic fluids and yield stress fluids is more intricate due to the incorporation of elastic forces or yield stress. We measured the surface tension of the six shower gel samples. It turns out that the three versathix- based samples almost have the same surface tension ( 26.5 [mN/m] ), and it is difficult to measure the surface tension of xanthan gum-based samples since they are yield stress fluid. We believe that the surface tension is not the dominant parameter for the rupture process in this work considering about the strong viscoelastic and yield stress behavior of the samples.

Fracture of viscoelastic fluids has been reported in many papers [11]. It has been understood that the process of fracture, characterized by its suddenness, is distinctly separate from smoother processes such as necking and disintegration driven by surface tension [15]. In this work, we believe that the xanthan gum-based samples are necking (ductile failure) as in Figure 1a–c, while the filament rupture process of versathix-based shower gel samples is sudden fracture as shown in Figure 1f.

The underlying physics of this study indeed holds fascinating prospects for further exploration. However, the primary aim of our present work was to develop a quick, economical emulative method based on image analysis. This method serves to quantify the commonly observed “gloppiness” phenomenon in complex fluids and gels. Delving into mechanisms beyond the observed “gloppy” behaviour will be a subject of our future work.

## 5. Conclusions

In this study, we have developed a cost-effective, high-speed emulative method based on image analysis to quantify the “gloppiness” level of shower gel samples. The degree of “gloppiness” determined by trained panelists exhibited clear inverse relationship with the rupture time ($t_{R}$) of the jetting flow.

The tested samples differ significantly in their rheological behavior. In fact, they represented the two main categories of personal care products in terms of their mechanical properties, sometimes referred to as “structured” and “non-structured”, i.e., having gel-like behavior with distinct yield stress and viscoelastic fluids with low-shear viscosity plateau. Nonetheless, we have shown that the same rupture time parameters can be applied to differentiate these samples in relation to the important sensorial attribute, namely gloppiness.

Gloppiness is a term used to describe the unique flow behavior and texture of certain complex fluids. It often refers to a semi-solid or thick liquid state that exhibits non-Newtonian flow properties, such as shear-thinning or yield stress. Gloppiness can be observed in materials like ketchup, mayonnaise, and certain polymer solutions, which display a combination of fluid and solid-like properties. Understanding and characterizing gloppiness is crucial for optimizing the processing and applications of these materials in various industries.

The study of rheological properties and flow dynamics in complex fluids plays a vital role in both academic and industrial areas. While pure shear and pure extensional rheological tests are significant in the study of complex fluids, they may not fully capture certain flow dynamics phenomena, such as “gloppiness”. A comprehensive understanding of these unique behaviors requires more advanced testing methods and analyses. Over the past few years, there has been an increasing utilization of computer vision and artificial intelligence technology to investigate the characteristics of complex fluids, including their rheology and fluid dynamics. An example of this trend is demonstrated by Mahmoudabadbozchelou and their team [16,17,18,19], who have published a series of studies outlining the implementation of machine learning techniques for the purpose of predicting and understanding the rheological behavior of complex fluids. The work of Maitrejean and colleagues, as outlined in their publication [20], showcases the synergistic application of computer vision and machine learning approaches to accurately identify the rheological properties of jetted fluids. Our belief is that the incorporation of machine learning and artificial intelligence will be a critical factor in the evolution of the cosmetics industry. In line with this, we are actively exploring the implementation of machine learning methods in our own project.

## Figures and Tables

**Table 1:** Shower gel formulations with versathix as the thickener.

Gloppiness Level	Low	Middle	High
Ingredient	wt%	wt%	wt%
DI water	84.32	84.12	83.92
Versathix	0.20	0.40	0.60
Other components	15.48	15.48	15.48

**Table 2:** Shower gel formulations with xanthan gum as the thickener.

Gloppiness Level	Low	Middle	High
Ingredient	wt%	wt%	wt%
DI water	71.900	71.500	71.000
Xanthan Gum	0.900	1.300	1.800
Other components	27.2	27.2	27.2

**Table 3:** Shower Gel parameters.

ID	$G_{0}$	Uneven Dispensing	Dispensing Slowness	Consistency	Holds Shape
SG-1	9.0	8.0	7.0	6.0	2.5
SG-2	8.0	8.5	9.0	6.0	2.5
SG-3	6.0	7.0	6.5	4.5	2.0
SG-4	5.0	5.5	7.0	6.0	7.0
SG-5	3.5	5.0	6.5	7.0	4.0
SG-6	2.0	3.0	3.0	2.0	7.0

**Table 4:** Maxium relaxation time ($\lambda_{s}$) of shower gel samples.

ID	$\lambda_{s}\left(s\right)$
Versathix-HG	0.12
Versathix-MG	0.15
Versathix-LG	0.16
XG-HG	/
XG-MG	/
XG-LG	/

## Data Availability

The data associated with the manuscript are available from the corresponding authors.
